# Transcranial direct current stimulation combined with physical or cognitive training in people with Parkinson’s disease: a systematic review

**DOI:** 10.1186/s12984-020-00701-6

**Published:** 2020-06-15

**Authors:** Victor Spiandor Beretta, Núbia Ribeiro Conceição, Priscila Nóbrega-Sousa, Diego Orcioli-Silva, Luana Karla Braz Fonseca Dantas, Lilian Teresa Bucken Gobbi, Rodrigo Vitório

**Affiliations:** 1 Institute of Biosciences, Posture and Gait Studies Laboratory (LEPLO), São Paulo State University (Unesp), Avenue 24-A, 1515, Bela Vista, Rio Claro, São Paulo State 13506-900 Brazil; 2 Graduate Program in Movement Sciences, São Paulo State University – UNESP, Rio Claro, São Paulo State Brazil; 3 Department of Neurology, Oregon Health and Science University, Portland, Oregon USA

**Keywords:** Neurodegenerative disease, Movement disorders, Transcranial stimulation, Physical therapy, Cognition, Rehabilitation

## Abstract

**Background:**

Pharmacologic therapy is the primary treatment used to manage Parkinson’s disease (PD) symptoms. However, it becomes less effective with time and some symptoms do not respond to medication. Complementary interventions are therefore required for PD. Recent studies have implemented transcranial direct current stimulation (tDCS) in combination with other modalities of interventions, such as physical and cognitive training. Although the combination of tDCS with physical and cognitive training seems promising, the existing studies present mixed results. Therefore, a systematic review of the literature is necessary.

**Aims:**

This systematic review aims to (i) assess the clinical effects of tDCS when applied in combination with physical or cognitive therapies in people with PD and; (ii) analyze how specific details of the intervention protocols may relate to findings.

**Methods:**

The search strategy detailed the technique of stimulation, population and combined interventions (i.e. cognitive and/or physical training). Only controlled studies were included.

**Results:**

Seventeen of an initial yield of 408 studies satisfied the criteria. Studies involved small sample sizes. tDCS protocols and characteristics of combined interventions varied. The reviewed studies suggest that synergistic effects may be obtained for cognition, upper limb function, gait/mobility and posture when tDCS is combined with cognitive and/or motor interventions in PD.

**Conclusion:**

The reported results encourage further research to better understand the therapeutic utility of tDCS and to inform optimal clinical use in PD. Future studies in this field should focus on determining optimal stimulation parameters and intervention characteristics for maximal benefits in people with PD.

## Introduction

Parkinson’s disease (PD) is a neurodegenerative disorder mainly characterized by the progressive loss of dopaminergic neurons in the substantia nigra pars compacta. This leads to excessive GABAergic (inhibitory) signaling from the output nuclei of the basal ganglia to the thalamus and other subcortical structures [1, 2]. In turn, the thalamus sends reduced excitatory signaling to many cortical areas, leading to a broad cortical dysfunction in PD [1, 2], which includes sensorimotor and cognitive areas. PD is traditionally described as a movement disorder, including symptoms such as bradykinesia, resting tremor, rigidity, postural instability and gait impairments [3, 4]. Mood disorders (e.g. anxiety and depressive symptoms) and cognitive impairments (e.g. executive function, memory, etc.) are also common and disabling in PD [5, 6]. Dopaminergic medication is the primary treatment used to manage PD symptoms. However, it becomes less effective and side effects emerge with time, such as dyskinesia, motor fluctuations and hallucination [7, 8]. Additionally, cognitive impairments and postural instability do not respond to dopaminergic medication [9, 10]. Complementary interventions are therefore required for PD.

A growing body of evidence suggests that transcranial direct current stimulation (tDCS), a low-cost method of non-invasive brain stimulation, could potentially become a clinical tool for PD in the near future [11–14]. tDCS directs, through scalp electrodes, a constant low amplitude electric current (generally between 1 and 2 mA), which has been shown to modulate excitability in both cortical [15] and subcortical brain areas [16, 17]. Anodal tDCS leads to increased neuronal excitability whereas cathodal tDCS leads to reduced neuronal excitability [15, 18]. tDCS can also modulate oxygen supply to cortical and subcortical areas [19] and neuronal synapsis strength [20], triggering plasticity processes. Of particular interest to its application in PD, anodal tDCS increases extracellular dopamine levels in the striatum [21] and inhibits GABAergic neurons [22, 23]. Recent systematic reviews confirmed that, as a stand-alone intervention, tDCS promotes benefits on motor function and to a lesser extent on cognition in people with PD [12, 24].

Taking advantage of tDCS portability, researchers have implemented tDCS in combination with other modalities of complementary interventions, such as physical (i.e. exercise, physiotherapy, etc.) [25] and cognitive training [26, 27]. The idea is that such combinations would promote greater, synergistic effects than the interventions applied separately [28, 29]. In this context, tDCS can be applied concurrently or as a priming technique. It has been argued that such applications may reinforce long-term potentiation-like processes [30], promoting greater retention of benefits from combined therapy [31]. Although the combination of tDCS with physical and cognitive training seems promising, the existing studies present mixed results. Therefore, a systematic review of the literature is necessary. This systematic review assessed: (i) the clinical effects of tDCS when applied in combination with physical or cognitive therapies in people with PD and; (ii) how specific details of the intervention protocols may relate to findings.

## Methods

### Search strategy

Two of the authors (VSB and NRC) created a search strategy, which was approved by all authors, to identify all potentially relevant studies. Table 1 shows the terms and synonyms used to search papers in the databases in the title, abstract or keywords. The search strategy included three fields (connected with “AND”) with independent search terms. Terms in the same field were connected with the conjunction “OR”. The first search field focused on the population (i.e. Parkinson’s disease). The second search field comprised types of non-pharmacologic treatments (i.e. motor/physical therapies, rehabilitation, cognitive therapies, and exercises). The third search field focused on tDCS. The search terms were combined and explored with the medical subject headings (MeSH) in different databases (Pubmed, Scopus, Embase, Web of Science and PsycNET). Manuscripts identified through databases search were downloaded to a reference manager software where duplicates were excluded. Two authors (VSB and NRC) performed the initial screen by reviewing the titles and abstracts and when necessary a third author (RV) made the final decision. However, in cases that the eligibility of the study was not clear by the information provided in the title and abstract, a review of the full text was performed. Additional sourced articles were acquired by screening reference lists.

**Table 1:** Search terms used in each search field

Population	Intervention	Transcranial stimulation
TITLE-ABS-KEY’	TITLE-ABS-KEY’	TITLE-ABS-KEY’
parkin^a^	Exercise	Transcranial direct current stimulation
	Training	tDCS
	Aerobic	transcranial electrical stimulation
	Anaerobic	
	Strength	
	“physical therapy”	
	“physical activity”	
	“physical program”	
	cue^a^	
	non-pharmacologic^a^	
	Fitness	
	Cognitive	
	Cognition	
	rehabilitation	
	Therapy	

^a^ indicates a wildcard; TITLE-ABS-KEY’ indicates a title, abstract and keyword search

### Inclusion and exclusion criteria

Articles were included if they investigated the effects of tDCS combined with physical and/or cognitive therapies on motor, cognitive, neuropsychiatric, quality of life, and others outcomes in people with PD (only human participants). Only articles written in English were considered for the review. Any open-label studies, review papers, book chapters, commentaries, study protocols, or clinical trials registers were excluded. Articles that analyzed the effects of tDCS as a stand-alone intervention and those involving other techniques of transcranial stimulation (e.g., transcranial alternating current stimulation, transcranial magnetic stimulation, etc.) were also excluded to avoid confusion with the reviewed topic.

### Data extraction

Data were extracted by five reviewers (VSB, NRC, PNS, DOS, LKBFD) and synthesized into a table format. Data entry was confirmed by another reviewer (RV). Data included authors, year of publication, groups and participants characteristics (number of participants, score of Unified Parkinson’s Disease Rating Scale motor section (UPDRS III), years since the diagnosis, levodopa equivalent daily dosage), study design, tDCS protocol (current stimulation, sham characteristics, electrode placement, stimulation intensity and duration, electrode size and number of sessions), therapy protocol (type, characteristics, volume, intensity, duration, moment and number of sessions), assessment (period; medication state and outcomes), main findings and occurrence of adverse effects of tDCS.

## Results

### Study selection

**Fig. 1:**
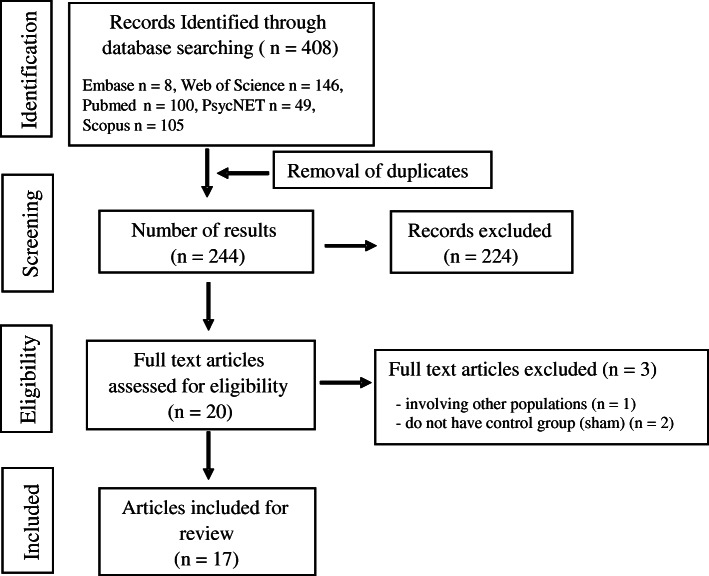
Flowchart with screening process following the PRISMA guidelines

The flow chart with information regarding the different phases of the search and screening process is shown in Fig. 1. The search strategy yielded 408 studies from publication databases. One hundred and sixty-four duplicates were removed. After further review of title and abstract, 20 articles were included by consensus of the reviewers. After full text review, three studies were excluded, one because it did not involve people with PD [32], and two because they did not have control group or sham condition [26, 27]. Table 2 shows the extracted data regarding the methodological aspects of the studies included in the present systematic review.

**Table 2:** Methodological characteristics of the studies included in the systematic review following the determination of the PICOS terms

**First Author (Year)**	**- Groups** (n = number of participants; mean ± standard deviations (year); UPDRS III (score); PD time (year); LED (mg/day)) • Study design	**tDCS** 1. Current Stimulation 2. Sham characteristics 3. Electrode place (anode/cathode) 4. Duration 5. Intensity 6. Electrode size (cm^2^) 7. Number of sessions	**Intervention** 1. Type (acute/chronic) 2. Characteristics (strength, gait, cognition, etc) 3. Volume (only chronic); Intensity; duration 4. Moment 5. Number of sessions	A. Assessment period B. Medication state C. Outcomes (methods/equipment)
Biundo (2015) [33]	- Cognitive Training + active tDCS (*n* = 12; 69.1 ± 7.6; NR; NR; NR) - Cognitive Training + sham tDCS (n = 12; 72.3 ± 4.1; NR; NR; NR) • Parallel, double-blind, randomized trial	1. Anodic 2. NR 3. Left DLPFC/contralateral supraorbital area 4. 20 min 5. 2 mA 6. NR 7. 16 sessions	1. Chronic 2. Computer-based cognitive training 3. 4 days a week; Rehacom software®; 30 min 4. NR 5. 16 sessions	A. Pre, post, and follow-up (16 weeks) B. NR C. Attention/executive skills (Written coding test); memory (immediate memory index and delayed memory index); disease severity (UPDRS III)
Broeder (2019) [28]	- tDCS + writing Parkinson group (*n* = 10; 63.2 ± 9.2; 17.5 range (13–22); 6.9 ± 5.1; 407 ± 300.4) • Cross-over, single-blind, randomized	1. Anodic 2. Current applied for the 30s 3. Left M1/ right supraorbital area 4. 20 min 5. 1 mA 6. 35 cm^2^ 7. 1 session	1. Acute 2. Writing 3. 3 bouts of writing several sequences of letters (3 min) followed by execution of the funnel task; 20 min 4. During tDCS 5. 1 session	A. Online effect B. ON state C. Number of upper limb freezing Episodes (funnel task on a touch-sensitive tablet)
Broeder (2019) [34]	- tDCS + writing Parkinson group (*n* = 10; 63.2 ± 9.2; 17.5 range (13–22); 7.0 ± 5.1; NR) • Cross-over, single-blind, randomized controlled trial	1. Anodic 2. NR 3. Left M1/Right supraorbital area 4. 20 min 5. 1 mA 6. 35 cm^2^ 7. 1 session	1. Acute 2. Writing 3. Writing of loops in different patterns (continuous and alternating) and sizes (0.6 and 1.0 cm) during 3 trials (2 min 24 s each) followed by execution of the funnel task (5 trials of 1 min each); 20 min 4. During tDCS 5. 1 session	A. Pre, during, post (30 min after training) and follow-up (1 week) B. ON state C. writing performance on tablet (amplitude, velocity, coefficients of variation); writing performance on paper (mean writing size, writing velocity and writing quality/Systematic Screening of Handwriting Difficulties test); motor cortex excitability – MEP, CSP, RMT and SICI (TMS)
Costa-Ribeiro (2016) [31]	- tDCS + gait training (*n* = 11; 61.1 ± 9.1; 19 ± NR; 6.1 ± 3.8; 740.9 ± 924.3) - Sham + gait training (*n* = 11; 62 ± 16.7; 19.1 ± NR; 6.3 ± 3.7; 890.9 ± 836) • Parallel, double-blind, randomized controlled trial	1. Anodic 2. Current applied for 30s 3. SMA/supraorbital area over the hemisphere of the most affected side 4. 13 min 5. 2 mA 6. 35 cm^2^ 7. 10 sessions	1. Chronic 2. Visually cued gait training (Subjects were instructed to walk at the step length indicated by white strips (visual cue) along a 6.5-m walkway) 3. 3 times a week; 24 min of active training, with a 6 min interval (30 min total) 4. After tDCS session 5. 10 sessions	A. Pre, post (48 h after training) and follow-up (1 month) B. ON and OFF state C. Functional mobility (TUG); motor cortex excitability – MEP (TMS)
Costa-Ribeiro (2017) [35]	- Cued gait training + tDCS (CGT + tDCS) (n = 11; 61.1 ± 9.1; 19.0 ± 4.9; 6.1 ± 3.8; 740.9 ± 924.3) - Cued gait training + sham (CGT + sham) (n = 11; 62.0 ± 16.7; 17.6 ± 5.1; 6.3 ± 3.7; 890.9 ± 836.0) • Parallel, double-blind controlled, randomized clinical trial	1. Anodic 2. The stimulator was turned off after 30 s 3. M1/supraorbital area of the contralateral hemisphere of the most affected side 4. 13 min 5. 2 mA 6. 35 cm^2^ 7. 10 sessions	1. Chronic 2. The gait training associated with visual cues was aimed to improve functional mobility 3. 3 days a week; NR; 30 min 4. After the tDCS 5. 10 sessions	A. Pre, post, and follow-up (1 month) B. ON state C. Functional mobility (TUG, 10-m walk test); Cadence, stride length (video camera); Motor Impairment (UPDRS III); Bradykinesia (sum of scores on UPDRS items 23–26 and UL-MT); Balance (BBS); Quality of life (PDQ-39)
Criminger (2018) [36]	tDCS (Sitting, Bike, Wii, Sham) (*n* = 16; 68.13 ± 9.76; 23.44 ± 9.73; 8.69 ± 9.76; NR) • Cross-over, single-blind, randomized controlled trial	1. Anodic 2. Current applied for 30s (1 to 0 mA) 3. Left DLPFC/ right DLPFC 4. 20 min 5. 2 mA 6. 15cm^2^ 7. 1 session	1. Acute 2. Bike/Wii (golf) 3. Bike: self-reported intensity level of 12–14 on the Borg Rating of Perceived Exertion Scale, Wii: NR; 20 min 4. During tDCS 5. 1 session	A. Post each session B. ON state C. Dual-task performance during walking (TUG and TUG with dual task (motor and cognitive task); dual-task cost- DTC.
Fernández-Lago (2017) [37]	- treadmill - treadmill + tDCS - treadmill + sham (*n* = 18; 56.67 ± 11.63; 21.17 ± 11.31; 6.17 ± 3.65; 733.2 ± 496.2^a^) • Cross-over, randomized	1. Anodic 2. 2 mA and turn off after 8 s 3. Motor cortex/ contralateral supraorbital area 4. 20 min 5. 2 mA 6. 3.5 cm^2^ 7. 1 session	1. Acute 2. Treadmill walking 3. 1 day a week; Individual velocity obtained during overground walking at the beginning of each experimental session (PRE) was used for the subsequent treadmill walking conditions; 20 min 4. During tDCS 5. 1 session	A. Pre and post each session B. ON state C. Gait (Optogait, Microgait); Neurophysiologic measurements: Electromyography, Reciprocal Ia Inhibition, H-reflex amplitude, MEP; SICI, ICF (EMG and TMS)
Forogh (2017) [38]	- tDCS + occupational therapy (*n* = 12; 61.33; NR; NR; NR) - Sham + occupational therapy (n = 11; 64.11; NR; NR; NR) • Parallel, double-blind, randomized clinical trial.	1. Anodic 2. Current applied for 30s 3. Left DLPFC/Right DLPFC 4. 20 min 5. 2 mA 6. 35 cm^2^ 7. 8 sessions	1. Chronic 2. Occupational therapy 3. 4 days a week (2 week); NR; NR. 4. After tDCS 5. 8 sessions	A. Pre, post and follow-up (3 months) B. NR C. Fatigue (Fatigue Severity Index); Daytime Sleepiness (Epworth Sleepiness scale)
Horiba (2019) [39]	- tDCS + mirror visual feedback (*n* = 9; 71.33 ± 4.15; 13 ± 5.56; 6.44 ± 3.16; 324.00 ± 121.11) - Sham + mirror visual feedback (n = 9; 70.67 ± 3.85; 17.11 ± 6.10; 6.44 ± 3.28; 251.56 ± 157.63) • Parallel, double-blind, randomized clinical trial.	1. Anodic 2. Current applied for 30s 3. Right M1/frontal orbit 4. 20 min 5. 2 mA 6. 80 cm^2^ 7. 1 session	1. Acute 2. Motor skill training using mirror visual feedback 3. 4 sessions of 30 s of execution and 30 s of rest for 5 min each session; observe the movements of the right hand in a mirror that provided mirror visual feedback of their performance in the ball rotation task; 20 min. 4. During tDCS 5. 4 sessions (1 day)	A. Pre and post B. OFF state C. Upper limb motor function (Number of ball rotations /video camera analysis, peak acceleration/infrared cameras and reflective markers, maximal pinching force), and disease severity (UPDRS III).
Ishikuro (2018) [40]	- Anodal tDCS + Physical therapy (n = 9; 77.5 ± 4.8; NR; 5.77 ± 2.03; NR) - Cathodal tDCS + Physical therapy - Sham tDCS + Physical therapy • Cross-over, randomized clinical trial.	1. Anodic and Cathodic 2. Current applied for 30s 3. Frontal polar area/occipital area 4. 15 min 5. 1 mA 6. 35 cm^2^ 7. 5 sessions	1. Chronic 2. Physical therapy for upper extremities (stretching and muscle strength exercise) while sitting in a chair 3. 5 days a week; 15 min 4. During tDCS 5. 5 sessions for each condition.	A. Pre, post 1 (1 session), post 2 (3 sessions) and post 3 (5 sessions). Post 1 and post 2 performed only for the STEF B. OFF state C. Disease severity (UPDRS III); Sensory-motor functions (Fugl Meyer Assessment set); Ability to pinch, grasp, and transfer objects (STEF); Executive function (TMT-A)
Kaski (2014) [25]	- tDCS + Physical training (*n* = 8; NR; NR; NR) - tDCS without physical training (n = 8; NR; NR; NR) • Cross-over, double-blind, randomized controlled trial	1. Anodic 2. The current (2 mA) was turned off after 30 s 3. Bilateral M1 and PMC/ inion 4. 15 min 5. 2 mA 6. 40 cm^2^ 7. 1 session	1. Acute 2. Gait initiation, stride length, gait velocity, arm swing, and balance 3. 15 min 4. During tDCS 5. 1 session	A. Pre and post B. ON state C. Gait (TUG, 6-min walk, and video analysis); Balance (Quantitative pull test, SwayStar System®, Balance Int. Innovations GmbH, Switzerland).
Kaski (2014) [41]	- Dance + tDCS - Dance + sham (1 person; 79; 34; 7; 856,25 + Piribedil 100 mg) • Cross-over, double-blind, randomized	1. Anodic 2. 2 mA for only 30s and then turned off 3.Bilateral M1 and PMC/ inion 4. 7 min 30 s 5. 2 mA 6. 40 cm^2^ 7. 2 sessions for each training (interval of 1 week)	1. Acute 2. Dance – tango 3. 2 music; 7 min 30 s 4. During tDCS 5. 2 sessions	A. Pre and post B. ON state C. Angular trunk movement during the dancing (digitally-based angular-velocity transducers); gait (Tinetti Gait Index)
Lawrence (2018) [42]	- Standard Cognitive Training (Standard CT) (*n* = 5; 68.14 ± 8.69; NR; 5.29 ± 4.23; 295 ± 313.40 - Tailored Cognitive Training (Tailored CT) (*n* = 6; 65.57 ± 5.20; NR; 5.79 ± 4.97; 383 ± 178.62 - tDCS (*n* = 7; 72 ± 6.45; NR; 5.50 ± 5.66; 573.29 ± 586.25 - Standard Cognitive Training + tDCS (Standard CT + tDCS) (n = 7; 63.57 ± 15.68; NR; 6.79 ± 4.60; 350.71 ± 322.37 - Tailored Cognitive Training + tDCS (Tailored CT + tDCS) (n = 7; 67.43 ± 6.37; NR; 4.43 ± 2.70; 464.29 ± 358.78 - Control Group (CG) (n = 6; 72.29 ± 6.21; NR; 5.36 ± 4.14; 292.88 ± 274.51 • Parallel, randomized controlled trial	1. Anodic 2. NR 3. Left DLPFC/ left supraorbital area. 4. 20 min 5. 1.5 mA 6. 35 cm^2^ 7. 4 sessions	1. Chronic 2. Cognitive training (Smartbrain Pro™): Standard (Two predetermined activities for each cognitive domain - memory, attention/working memory, language, executive function, visuospatial) or Tailored (activities individualized to participants baseline neuropsychological test results). 3. 3 days a week; difficulty levels of each activity were adjusted individually; 45 min 4. Separated of tDCS session 5. 12 sessions	A. Pre, post (week 5), and follow-up (week 12) B. ON state C. Neuropsychological assessment (executive function, attention - working memory, memory, visuospatial abilities, language, global cognitive, activities of daily living and quality of life)
Manenti (2016) [29]	- Physical therapy + tDCS (*n* = 10; 69.0 ± 9.1; 27.8 ± 13.9; 7.1 ± 3.6; 524.6 ± 179.1) - Physical therapy + sham (n = 10; 69.1 ± 5.6; 27.6 ± 8.9; 7.8 ± 4.2; 815.7 ± 590.9) • Parallel, double-blind, randomized	1. Anodic 2. 2 mA for only 10s and then turned off and turned on in the last 10s 3. DLPFC contralateral to the most affected body side/contralateral supraorbital area 4. 25 min 5. 2 mA 6. 35 cm^2^ 7. 10 sessions	1. Chronic 2. Focused on the core areas of motor impairment in PD, such as the inability to initiate movement, difficulties with balance and gait control, falls, and deficits in the pacing of rhythmic movements. 3. 5 days a week; 25 min 4. During tDCS 5. 10 sessions	A. Pre, post, and follow-up (3 months) B. ON state C. Cognition (MMSE, PD-CRS, Digit span, Cantab Paired Associated Learning, TMT, FAB, Semantic fluency, Cantab Reaction Time Index); clinical evaluation (UPDRS-III, HY, BDI-II, PDQ-39, RBDSQ); motor function (TUG, Four Square Step Test, Standing Stork Test, Sit and Reach Test)
Manenti (2018) [43]	- tDCS + computerized cognitive training (*n* = 11; 65.5 ± 6.4; 26 ± 10.3; 6.2 ± 3.9; 618.6 ± 304.4) - Sham + computerized cognitive training (n = 11; 63.8 ± 7.1; 22.7 ± 7.8; 7.6 ± 3.4; 559.8 ± 306.5) • Parallel, double-blind, randomized	1. Anodic 2. 2 mA for only 10s and then turned off and turned on in the last 10s 3. left DLPFC/right supraorbital area 4. 25 min 5. 2 mA 6. 35 cm^2^ 7. 10 sessions	1. Chronic 2. BrainHQ (Posit Science) – exercise focused on attention, memory, brain speed, people skills, navigation and intelligence. Five exercises of 5 min for each session. 3. 5 days a week for 2 weeks; 25 min 4. During tDCS 5. 10 sessions	A. Pre, post and follow-up (3 months) B. ON state C. Clinical and disease severity (PDQ-39, BIS-11, RBDSQ, Apathy Evaluation Scale, UPDRS III, H&Y); Cognitive functions (MMSE; PD-CRS, Digit span, Rey Auditory Verbal Learning Test, Object and action IPNP, TMT, FAB, phonemic and Semantic fluency, Stroop); depressive symptoms (BDI-II)
Schabrun (2016) [44]	- Active tDCS + dual-task gait training (*n* = 8; 72 ± 4.9; 47.7 ± 7.5; 6.9 ± 4.4; 730 ± 341 - Sham + dual-task gait training (n = 8; 63 ± 11.0; 37.7 ± 9.8; 4.6 ± 3.9; 523 ± 398 • Parallel, double-blind, randomized, sham-controlled	1. Anodic 2. Ramped up over 10 s, down over 10 s and then switched off. 3. M1/contralateral supraorbital area 4. 20 min (In the first 20 min of Dual-task gait training) 5. 2 mA 6. 35 cm^2^ 7. 9 sessions	1. Chronic 2. Gait training + cognitive task 3. 3 days a week; progressive complexity; 60 min 4. During tDCS 5. 9 sessions	A. Pre, Post, and follow-up (12 weeks) B. ON state C. Gait, Gait + cognitive task (GAITRite® and TUG); bradykinesia (clinical test); visuomotor speed and procedural learning (Serial Reaction Time Task).
Yotnuengnit (2018) [45]	- tDCS (*n* = 18; 64.4 ± 7.8; 10,89 ± 4,75; 7,9 ± 3,9; 849,1 ± 397,1) - tDCS + physical therapy (*n* = 17; 68.2 ± 9.8; 11,94 ± 4,68; 9,4 ± 5,3; 829,0 ± 360,6) - Sham + physical therapy (n = 18; 62.7 ± 2.8; 11,17 ± 3,97; 6,6 ± 3,6; 912,0 ± 472,9) • Parallel, double-blind, randomized controlled trial	1. Anodic 2. 2 to 0 mA in the first minute 3. M1/ right supraorbital area 4. 30 min 5. 2 mA 6. 35 cm^2^ 7. 6 sessions	1. Chronic 2. Joint range of motion and body flexibility, strengthening leg muscles, balance and gait training 3. 3 days per week; 30 min 4. After tDCS 5. 6 sessions	A. Pre, Post, and follow-up (2 and 6 weeks) B. ON state C. Gait (The Gait & Motion Analysis); Disease severity (UPDRS)

^a^LED was calculated according to Tomlinson et al. (2010) [46]; PD = Parkinson’s disease; UPDRS III = motor part of Unified Parkinson’s disease rating scale; LED = Levedopa equivalent dose; tDCS = transcranial direct current stimulation; NR = not reported; M1 = primary motor cortex; PMC = pre-motor cortex; DLPFC = Dorsolateral Prefrontal Cortex; TUG = Timed Up and Go Test; MMSE = Mini-Mental State Examination; PD-CRS = Parkinson’s Disease Cognitive Rating Scale; TMT = Trial Making Test; HY = Hoehn and Yahr Scale; BDI-II = Beck Depression Inventory-II; PDQ-39 = Parkinson’s Disease Quality of Life Questionnaire-39; RBDSQ = REM Sleep Behavior Disorders Screening Questionnaire; SICI = short intracortical inhibition; MEP = Motor evoked potential; ICF = Intracortical facilitation; UL-MT = upper limb motor task; BBS = Berg Balance Scale; EEG = electroencephalography; DTC = dual-task cost; TMS = Transcranial magnetic stimulation; EMG = electromyography; CSP = cortical silent period; RMT = resting motor threshold; STEF = simple test for evaluating hand function; FAB = Frontal Assessment Battery; BIS-11 = Barratt Impulsivity Scale; IPNP = International Picture Naming Project

### Participants

The sample size varied from 1 to 53 participants, with a mean age between 56.67 and 79 years. The mean of the UPDRS motor section (part III) score ranged from 10.9 to 47.7. The disease duration ranged from 4.4 to 9.4 years and Levodopa Equivalent Dose (LED) ranged from 251.56 to 912 mg/day.

### Study design

Seven studies had cross-over design [25, 28, 34, 37, 39–41] and ten were controlled trials with parallel arms. Three studies did not mention information regarding how or if experiments were blinded [37, 40, 42], three studies consisted of single-blind experiments [28, 34, 36] and eleven studies consisted of double-blind experiments. It is worth mentioning that all the included studies randomly assigned participants into groups/conditions.

### tDCS protocol

#### Polarity, current intensity, and number of sessions

Fifteen studies included anodal tDCS protocols targeting a single brain region [28, 29, 31, 33–40, 42–45] whereas two studies stimulated both hemispheres [25, 41]. Thirteen studies used 2 mA [25, 29, 31, 33, 35–39, 41, 43–45], one study used 1.5 mA [42] and three studies used 1 mA [28, 34, 40]. Seven studies used a single session of tDCS protocols [25, 28, 34, 36, 37, 39, 41] and ten studies applied multiple sessions of tDCS protocols, varying between 2 and 16 sessions.

#### Electrode size and placement

Fifteen studies used 35–40 cm^2^ electrodes, one study used 15 cm^2^ electrodes [36] and one study used 80 cm^2^ electrodes [39]. Anodal electrode was placed over the dorsolateral prefrontal cortex (DLPFC) in six studies [29, 33, 36, 38, 42, 43], frontal polar area in one study [40] and over motor areas (i.e., primary motor cortex (M1), premotor cortex (PMC) and supplementary motor area (SMA)) in ten studies [25, 28, 31, 34, 35, 37, 39, 41, 44, 45]. The reference electrode (cathode) was placed over the contralateral supraorbital region in twelve studies [28, 29, 31, 33–35, 37, 38, 43–45], over the ipsilateral supraorbital region in one study [42], over the frontal orbit in one study [39], over the inion in two studies [25, 41] and over the occipital area in one study [40].

#### Duration of the stimulation (active and sham)

Stimulation time for active protocols varied between 7.5 and 30 min, with nine studies using 20-min sessions [28, 33, 34, 36–39, 42, 44]. Most of the studies used sham protocols in which the current was delivered during the initial period of the session (8 to 60 s) and then turned off; ten studies reported that the current was delivered during the initial 30 s [25, 28, 31, 35–41]. Two studies reported that the current was turned on once again for the last 10 s of the session [29, 43].

### Combined interventions

tDCS protocols were combined with motor interventions in thirteen studies [25, 28, 29, 31, 34–41, 45], cognitive interventions in three studies [33, 42, 43] and motor-cognitive intervention in one study [44].

Seven studies combined tDCS protocols with acute interventions [25, 28, 34, 36, 37, 39, 41] and in all of them tDCS was applied concurrently (not as a priming technique). Ten studies combined tDCS protocols with chronic interventions [29, 31, 33, 35, 38, 40, 42–45]. Five of the chronic studies applied tDCS as a priming technique [31, 35, 38, 42, 45] while five studies applied tDCS during the combined intervention [29, 33, 40, 43, 44]. Chronic interventions varied between 3 and 5 sessions per week (between 5 and 16 sessions in total), with total duration (not only tDCS) between 15 and 60 min [29, 31, 33, 35, 38, 40, 42–45].

### Assessment characteristics

Fifteen studies carried out pre- and post-assessments [25, 29, 31, 33–35, 37, 38, 41, 42, 44, 45] and seven chronic studies also included follow-up assessments after 4 [31, 35] or 12 weeks [29, 38, 42–44]. Twelve studies assessed patients while ON medication [25, 28, 29, 34–37, 41–45], two studies while OFF medication [39, 40], one study assessed patients in both ON and OFF states [31] and two studies did not report medication state [33, 38]. Although the methods used for assessment varied in the included studies, a few tests were repeated. The Timed Up and Go test (TUG) was used in six studies [25, 29, 31, 35, 36, 44], the UPDRS motor section was used in seven studies [29, 33, 35, 39, 40, 43, 45], Parkinson’s Disease Quality of Life Questionnaire-39 (PDQ-39) was used in four studies [29, 35, 42, 43] and Mini Mental State Examination (MMSE) and Parkinson’s Disease – Cognitive Rating Scale (PD-CRS) were used in three studies [29, 42, 43].

### Effects of tDCS

**Fig. 2:**
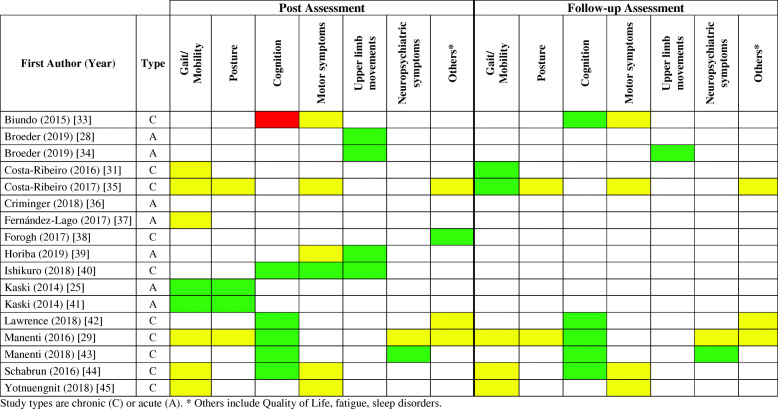
Synthesis of results in relation to the additional effect of tDCS. Green = Additional effect of combined intervention; Yellow = No additional effect of the combined intervention; Red = Negative effect of the combined intervention; White = not assessed in the reviewed studies; A = Acute (Considered the immediate effect of a single session); C = Chronic (Considered the effect of repeated sessions). * Others include Quality of Life, fatigue, sleep disorders

Table 3 presents the main results regarding the effects of tDCS when combined with motor and/or cognitive interventions. Figure 2 summarizes the main findings of the 17 included studies separately for gait/mobility, postural control, upper limb movements, other motor symptoms, cognition, neuropsychiatric symptoms and others (including quality of life, fatigue and sleep disorders).

**Table 3:** Main results of the reviewed studies

First Author (Year)	* Main Results
	• Adverse effects (occurrence)
Biundo (2015) [33]	* Active tDCS reduced performance in the attention/executive skills and delayed memory index when compared to sham tDCS at the post-test.
	* Active tDCS tends to improve performance in the immediate memory index compared to the sham group at the follow-up test.
	* No significant UPDRS-III motor changes were observed between groups at 4 and 16-week follow-up tests.
	• NR.
Broeder (2019) [28]	* Active tDCS decreased the episodes of freezing compared to sham tDCS.
	* No effects of tDCS were found for the amplitude, variability, and speed of the strokes outside the freezing episodes.
	* Patients who reported freezing episodes in daily life (n = 6) showed a beneficial effect of tDCS on stroke characteristics.
	• No adverse events of tDCS were reported.
Broeder (2019) [34]	* Active tDCS improved writing during the tDCS protocol, at the post-test and at follow-up compared to sham. * Active tDCS increased writing amplitude at follow-up period compared to post period. * Active tDCS enhanced cortical excitability compared to sham at the post-test.
	* Active tDCS enhanced cortical excitability compared to sham at the post-test.
	• No adverse events of tDCS were reported.
Costa-Ribeiro (2016) [31]	* Both groups improved functional mobility either in on or off medication condition compared with baseline.
	* However, for both medication conditions, these gains were maintained only in the tDCS+ gait training at follow-up test.
	* In the tDCS + gait training, enhancement of cortical excitability was observed at post-intervention and 1-month follow-up (both only for the “on” phase).
	• NR.
Costa-Ribeiro (2017) [35]	* Both groups improved functional mobility (velocity, cadence, and TUG), motor impairment, bradykinesia, balance, and quality of life at post-test.
	*For all outcome measures, no significant differences were found between groups.
	* The improvement in velocity and TUG were maintained at the follow-up test only for patients in the Cueing gait training + tDCS group.
	• No adverse events were reported by any of the participants.
Criminger (2018) [36]	*No differences were observed for TUG between conditions.
	*Increased DTC in the TUG motor (gait) after a tDCSbike session when compared to tDCSwii.
	*Increased DTC in the TUG cognitive (cognitive) after a tDCSbike session when compared to tDCSwii.
	*Increased DTC in the TUG cognitive (gait) after a tDCSwii session when compared to tDCSbike.
	• 1 participant was excluded from the initial sample (*n* = 18) after presenting headache in the first session.
Fernández-Lago (2017) [37]	* All groups increased velocity, stride length, and short intracortical facilitation at post-test.
	* All groups decreased Hmax/Mmax ratio and intracortical facilitation at post-test.
	* Sham tDCS + treadmill and treadmill groups decreased reciprocal Ia inhibition at post-test when compared to pre-test.
	• NR.
Forogh (2017) [38]	* Active tDCS + occupational therapy improved fatigue at post-test when compared to baseline. • NR.
Horiba (2019) [39]	* tDCS + mirror visual feedback increased the number of ball rotations at post-test. * No significant changes on UPDRS-III motor section were observed. • NR.
Ishikuro (2018) [40]	* Anodal tDCS decreased normalized scores of disease severity (UPDRS III) compared with Sham and Cathodal stimulation. * Anodal stimulation improved executive function and increased normalized scores of sensory-motor functions compared with Sham stimulation. * Anodal stimulation increased normalized scores of STEF compared with Cathodal stimulation. • 55.6% felt mild tingling. No adverse events were reported by any of the participants.
Kaski (2014) [25]	* tDCS + physical training increased gait velocity and stride length when compared with tDCS.
	* tDCS + physical training decreased the walking time and the time taken to regain stability following the retropulsion stimulus when compared with tDCS.
	* tDCS + physical training improved the turn phase of TUG.
	* Sham + physical training decreased walking time and increased stride length but these were comparatively less than with tDCS + physical training.
	• NR.
Kaski (2014) [41]	* Dance + tDCS increased peak trunk velocity in both pitch and roll directions.
	* Dance + tDCS increased for the 90% velocity range and total trunk velocity area.
	* Dance + tDCS increased gait function.
	• NR.
Lawrence (2018) [42]	* Standard CT improved memory at follow-up test, quality of life, and activities of daily life at post-test. However, decreased visuospatial ability at follow-up test.
	* Tailored CT improved attention/working memory at follow-up and quality of life at post- and follow-up tests.
	* tDCS improved attention/working memory at post- and follow-up tests, and memory at post-test.
	* Standard CT + tDCS improved executive function and attention/working memory at post and follow-up tests, and language and quality of life at post-test.
	* Tailored CT + tDCS improved executive function, memory, and language at post- and follow-up tests, and attention/working memory at the follow-up test.
	* CG had no improvements.
	• NR.
Manenti (2016) [29]	* Both groups showed improvement in depression at post- and follow-up tests.
	* Physical therapy + tDCS increased PD-CRS total, frontal-subcortical scores and verbal fluency at post, and stabilized the effect at follow-up test.
	* Physical therapy + tDCS group decreased the time necessary for completing TMT-B at post-test.
	* Both groups improved the Standing Stork, Four Square Step, and Sit, and Reach Tests at post-test, with improvements maintained at follow-up test for the Standing Stork, Four Square Step tests.
	* Both groups improved TUG performance at follow-up test.
	• NR.
Manenti (2018) [43]	* Both groups improved language, attentional and executive functions at post and follow-up periods. * Both groups increased phonemic fluency at post-test and semantic fluency at follow-up. * tDCS + computerized cognitive training showed lower depressive symptoms and greater phonemic fluency when compared to Sham + computerized cognitive training at post-test and follow-up. • No adverse events were reported by any of the participants.
Schabrun (2016) [44]	* Both groups improved gait velocity, cadence, step length and double support time in gait dual-tasks and bradykinesia at post- and follow-up tests.
	* Both groups improved functional mobility during TUG with words at post and follow-up tests.
	* Active tDCS + dual-task gait training improved the number of correct responses during TUG with counting and TUG with words at post-test, with a trend to maintain this performance in TUG with words at follow-up test.
	* There were no differences between groups for reaction time and attention.
	• One participant experienced strong tingling over the site of one electrode and a momentary flash of light in his eyes. The sensations lasted approximately 5 s. The participant ceased training that day but continued on subsequent days with no other events, and no other symptoms.
Yotnuengnit (2018) [45]	* All groups improved gait velocity and step time at post-test and at 2nd and 6th week follow-up.
	* Physical therapy group increased cadence at 2nd and 6th week follow-up tests.
	* tDCS and sham + physical therapy improved UPDRS II in all tests and the tDCS + physical therapy improved at the post and 2 weeks follow tests.
	* All groups improved UPDRS III at post and 2nd week follow-up tests.
	• Burning sensation (tDCS group).

PD = Parkinson’s disease; UPDRS III = motor part of Unified Parkinson’s disease rating scale; tDCS = transcranial direct current stimulation; NR = not reported; TUG = Timed Up and Go Test; CT = cognitive training; CG = control group; PD-CRS = Parkinson’s Disease Cognitive Rating Scale; TMT = Trail Making Test; DTC = dual-task cost; Hmax = maximum H-reflex amplitude; Mmax = maximum M amplitude; STEF = simple test for evaluating hand function; * indicate the main results; • indicate the adverse effects (occurrence)

#### Gait and mobility

Two out of eight studies reported synergistic effects on gait and mobility at post-test, including increases in gait speed and stride length and improvement in the turn phase of TUG [25, 41]. The other six studies assessing gait reported similar findings for both active and sham tDCS at post-test [29, 31, 35, 37, 44, 45]. Two of out five studies observed synergistic effects on gait and mobility at follow-up assessment [31, 35]. The other three studies assessing gait and mobility reported similar findings for both active and sham tDCS at follow-up test [29, 44, 45].

#### Postural control

Two out of four studies reported synergistic effects on postural control at post-test, including reduced time taken to recover balance following retropulsion and increased trunk peak velocity during tango [25, 41]. The other two studies assessing postural control reported similar findings for both active and sham tDCS at post-test and follow-up test [29, 35].

#### Upper limb movements and motor symptoms

The four studies assessing upper limb function observed synergistic effects. Broeder et al. (2019) [28] observed that active tDCS reduced the number of upper limb freezing episodes during writing. Synergistic effects were demonstrated for upper limb movement at post-test and follow-up [34, 39, 40]. Five out of six studies assessing motor symptoms and/or disease severity throughout clinical test reported similar findings for both active and sham tDCS at post-test and follow-up [33, 35, 39, 44, 45]. Only one study reported synergistic effects on disease severity at the post-test [40].

#### Cognition

Five out of six studies reported synergistic effects on cognition at post-test, including increased number of correct responses during the TUG with dual task, improved executive function, attention, working memory, verbal fluency and the total and frontal-subcortical scores on PD-CRS [29, 40, 42–44]. The benefits offered by the combined interventions on cognition were maintained at the follow-up test. On the other hand, Biundo et al. (2015) [33] observed negative effects of tDCS on cognition when combined with cognitive training. These authors reported decrement performance for the active tDCS compared to the sham group in attention/executive skills at post-test; additionally, only the sham group improved delayed memory index at post-test. Interestingly, Biundo et al. (2015) [33] observed a trend for better performance in the active tDCS group compared with the sham group in the story learning test at follow-up.

#### Neuropsychiatric symptoms, fatigue, sleep disorders and quality of life

Neuropsychiatric symptoms (i.e. depression [29]), sleep disorders [38] and/or quality of life [29, 35, 42] were less frequently assessed. Synergistic effects on neuropsychiatric symptoms (at post-test and follow-up) [43] and fatigue (at post-test) [38] were reported by only one study.

## Discussion

This review examined 17 studies that assessed the effects of tDCS when applied in combination with physical or cognitive therapies in people with PD. In summary, the included studies had appropriate design (i.e. cross-over or parallel arms) and control (i.e. sham tDCS). However, most included studies involved small sample sizes (*n* < 24), which makes the results difficult to generalize to the full range of people with PD. Also, tDCS protocols varied for stimulation time (from 7.5 and 30 min) and number of sessions (from 1 to 16 sessions), making comparisons and definitive conclusion regarding potential synergistic effects challenging. The most consistent synergistic effects were reported for cognition [29, 33, 40, 42–44] and upper limb function [28, 34, 39, 40]. Although findings related to other aspects of PD were inconsistent, synergistic effects were also reported for gait and postural control [25, 31, 35, 41]. The large heterogeneity in stimulation parameters and combined interventions may explain the large variation of findings that have been reported by the reviewed studies. In addition, it is not possible to determine if clinical and demographic characteristics of the studied individuals influenced the observed variability on results.

### Methodological aspects

The included studies were consistent with regards to tDCS polarity and site of stimulation. In all studies, the anode electrode was placed in order to target brain area related to motor or cognitive functions. Studies aiming to improve cognition targeted DLPFC while those aiming to improve motor aspects of PD targeted M1, SMA and/or PMC. As PD is characterized by reduced dopaminergic signaling by the substantia nigra pars compacta and the consequent increased GABAergic signaling from the basal ganglia to other encephalic regions, it makes sense to use anodal tDCS for rehabilitation in PD. Anodal tDCS has been shown to increase extracellular dopamine levels in the striatum [21] and inhibit GABAergic neurons [22, 23]. However, it is somewhat surprising that only one of the reviewed studies used cathodal tDCS [40]. Ishikuro et al. [40] applied the cathodal tDCS over the occipital area. A growing body of evidence supports the hypothesis that the functional interhemispheric imbalance contributes to the clinical motor deficits in PD. For example, PD is associated with asymmetry in M1 excitability [47, 48], with the more-affected hemisphere showing decreased excitability in comparison to the less-affected one. Thus, cathodal tDCS applied to the less-affected hemisphere (as well as anodal tDCS on the more-affected hemisphere) may also benefit patients with PD by leading to a more balanced interhemispheric activity. Cosentino et al. (2017) [49] observed that anodal tDCS of the more-affected M1 and cathodal tDCS of the less-affected M1 were able to induce polarity-specific changes in cortical excitability, leading to a more balanced interhemispheric excitability. These authors also observed that motor performances of both hands improved after both stimulation protocols [49]. Additional studies investigating the effects of cathodal tDCS in PD is required, especially when applied in combination with physical or cognitive interventions.

Another consistent aspect of the intervention protocols of the reviewed studies refers to current intensity. Thirteen studies used 2 mA and none compared the effects of different tDCS intensities when applied in combination with other interventions. The choice for 2 mA may be justified by the fact that some neurophysiological studies have shown greater increase in cortical excitability after 2 mA tDCS when compared to 1 mA [50, 51]. Also, longer lasting effects have been associated with greater current intensities [15]. However, other studies have found no differences in cortical excitability when comparing 2 mA and 1 mA [52–54]. Further research is required to understand if current intensity is a moderator factor when tDCS is applied in combination with other complementary interventions. Tolerability and safety of current intensities greater than 2 mA are still to be investigated in this kind of interventions in PD.

### Reported findings

Consistent synergistic effects were reported for cognition when tDCS was applied in combination with other modalities of complementary interventions. All six studies reporting synergistic effects on cognition used multiple sessions (4 to 16 sessions). Current intensity included 1.0, 1.5 or 2.0 mA. The reported synergistic effects were consistent with the area targeted by tDCS. Four studies targeted the DLPFC and one study target the frontal polar area, cortical regions known to be involved in executive function and working memory [29, 33, 42]. Interestingly, one study that stimulated M1 observed increased number of correct responses provided while performing the TUG test under dual-task condition (i.e., concomitant cognitive task) [44]. Authors argued that individuals with PD improved their ability to dual task when walking due to improved movement automaticity after the intervention. It is possible that anodal tDCS on M1 improved the efficiency of the direct locomotor pathway (i.e. neuronal commands are transmitted directly from M1 to the spinal cord), leading to a more automatic control [55, 56]. Then, the attentional and executive resources previously required for the control of movements could be reallocated to the performance of the concomitant cognitive task, which led to better performance of such task.

Upper limb function and motor symptoms (as assessed by UPDRS-III) respond differently to combined interventions involving tDCS. Consistent synergistic effects were reported for upper limb function [28, 34, 39, 40]. Since methods of the studies reporting the synergistic effects on upper limb function varied, it is difficult to establish associations with results. Overall, synergistic effects were observed after a single or multiple sessions, with tDCS targeting DLPFC or M1. On the other hand, motor symptoms as assessed by UPDRS-III seem to not benefit from the addition of tDCS on physical/cognitive interventions. Only one (out of five) study observed synergistic effects on motor symptoms [40]. UPDRS-III may miss subtle motor improvements and, therefore, we suggest future studies to use more objective assessments of clinical motor symptoms (e.g., inertial sensors, electromyography, etc.).

Although inconsistent, synergistic effects were also reported for gait and mobility when tDCS was combined with other complementary interventions in people with PD. Two out of eight studies reported synergistic effects of tDCS on gait and mobility at post-test [25, 41]. These two studies involved immediate physical interventions (i.e. gait training and tango dance) with stimulation targeting bilateral M1 and PMC. Studies targeting one cortical area (i.e. unilateral M1 or prefrontal cortex - PFC) observed similar results for both active and sham tDCS. Given the multiple cortical regions involved in gait and the bilateral representation of these regions [55, 57], it is possible that bilateral stimulation of multiple cortical areas is required to provide synergistic immediate effects (i.e. at post-test) to gait in people with PD. Also, two studies observed synergistic effects on gait and mobility at follow-up assessment [31, 35]. Costa-Ribeiro et al. [31, 35] observed that tDCS (applied before the physical intervention) prolonged the effects of cued gait training on functional mobility and that this benefit is independent of dopaminergic medication. The synergistic effects on mobility at follow-up assessment could be explained by the changes in cortical excitability. While both sham and active tDCS groups increased cortical excitability and improved mobility at post assessment, only the gait training plus active tDCS group maintained the benefits at the follow-up assessment [31]. Thus, there seems to be a positive relationship between increased cortical excitability and improvement in mobility in patients with PD [11, 31].

Synergistic effects on postural control were also reported when tDCS was combined with physical interventions in people with PD. Four studies assessed postural control, but only two studies presented synergistic effects of active tDCS combined with gait training and tango dance [25, 41]. These studies involved a single session of intervention and stimulated motor areas (i.e. M1 and PMC) bilaterally. The other two studies that reported no synergistic effects at the post and follow-up assessments involved 10 sessions of physical interventions (i.e. physical therapy and cued gait training) and stimulated M1 and PFC unilaterally (contralateral to the most affected body side). Postural control involves several cortical areas (i.e. PFC, PMC, SMA, M1, and primary sensory cortex – S1) in both hemispheres and the cerebellum [2, 58]. Therefore, it is possible that bilateral stimulation of multiple areas is necessary for the synergistic immediate effects to postural control in patients with PD. Besides, it should be noted that the synergistic effects were evidenced by studies that performed the stimulation during the physical intervention. Physical training has been shown to normalize M1 excitability in people with PD [59], while tDCS may decrease the threshold for these changes to occur, facilitating long-lasting effects [11, 25].

Limitations apply while interpreting the current findings. This review is limited by the small number of papers identified (*n* = 17) in the literature and the varied protocols tested in the included studies. This limits our interpretations and makes definitive conclusion regarding potential synergistic effects challenging. Additionally, although we acknowledge the contributions of open-label studies to the developments in this emerging area of research, we opted to exclude open-label studies from our review due to the inherent methodological flaws of such design. Despite these limitations, this review provides a useful synthesis of the existing studies on the combination of tDCS with physical/cognitive interventions in PD, which may guide the development of the field towards a more robust body of evidence.

## Conclusions

Although the reviewed studies used appropriate design and control, they involved a limited number of participants, which may imply underpowered analysis. Thus, large-scale studies are needed. Despite this major flaw, the reported results of tDCS interventions combined with cognitive and/or motor interventions encourage further research to better understand its therapeutic utility and to inform optimal clinical use in PD. The reviewed studies suggest that synergistic effects may be obtained for cognition, upper limb function gait/mobility and posture when tDCS is combined with cognitive and/or motor interventions in PD. Future studies in this field should focus on determining optimal stimulation parameters and intervention characteristics for maximal benefits in people with PD. Research on identifying potential predictors of response to tDCS-based interventions (i.e. tDCS combined with cognitive and/or motor interventions) in people with PD should also be conducted.

## Data Availability

All primary data were extracted from the referenced sources. The datasets used during the current study are available from the corresponding author on reasonable request.

## Abbreviations

PD: Parkinson’s disease
tDCS: Transcranial direct current stimulation
MeSH: Medical subject headings
UPDRS: Unified Parkinson’s Disease Rating Scale
LED: Levodopa Equivalent Dose
DLPFC: Dorsolateral prefrontal cortex
M1: Primary motor cortex
PMC: Premotor cortex
SMA: Supplementary motor area
PFC: Prefrontal cortex
S1: Primary sensory cortex
TUG: Timed Up and Go test
PDQ-39: Parkinson’s Disease Quality of Life Questionnaire-39
MMSE: Mini Mental State Examination
PD-CRS: Parkinson’s Disease – Cognitive Rating Scale
mA: Milliamp
NR: Not reported
TMT: Trial Making Test
HY: Hoehn and Yahr Scale
BDI-II: Beck Depression Inventory-II
RBDSQ: REM Sleep Behavior Disorders Screening Questionnaire
SICI: Short intracortical inhibition
MEP: Motor evoked potential
ICF: Intracortical facilitation
UL-MT: Upper limb motor task
BBS: Berg Balance Scale
EEG: Electroencephalography
DTC: Dual-task cost
TMS: Transcranial magnetic stimulation
EMG: Electromyography
CT: Cognitive training
CG: Control group
Hmax: Maximum H-reflex amplitude
Mmax: Maximum M amplitude
CSP: Cortical silent period
RMT: Resting motor threshold
STEF: Simple test for evaluating hand function
FAB: Frontal Assessment Battery
BIS-11: Barratt Impulsivity Scale
IPNP: International Picture Naming Project
